# Password-Only Authenticated Three-Party Key Exchange with Provable Security in the Standard Model

**DOI:** 10.1155/2014/825072

**Published:** 2014-04-14

**Authors:** Junghyun Nam, Kim-Kwang Raymond Choo, Junghwan Kim, Hyun-Kyu Kang, Jinsoo Kim, Juryon Paik, Dongho Won

**Affiliations:** ^1^Department of Computer Engineering, Konkuk University, 268 Chungwondaero, Chungju, Chungcheongbukdo 380-701, Republic of Korea; ^2^Information Assurance Research Group, Advanced Computing Research Centre, University of South Australia, Mawson Lakes, SA 5095, Australia; ^3^Department of Computer Engineering, Sungkyunkwan University, 2066 Seoburo, Suwon, Gyeonggido 440-746, Republic of Korea

## Abstract

Protocols for password-only authenticated key exchange (PAKE) in the three-party setting allow two clients registered with the same authentication server to derive a common secret key from their individual password shared with the server. Existing three-party PAKE protocols were proven secure under the assumption of the existence of random oracles or in a model that does not consider insider attacks. Therefore, these protocols may turn out to be insecure when the random oracle is instantiated with a particular hash function or an insider attack is mounted against the partner client. The contribution of this paper is to present the first three-party PAKE protocol whose security is proven without any idealized assumptions in a model that captures insider attacks. The proof model we use is a variant of the indistinguishability-based model of Bellare, Pointcheval, and Rogaway (2000), which is one of the most widely accepted models for security analysis of password-based key exchange protocols. We demonstrated that our protocol achieves not only the typical indistinguishability-based security of session keys but also the password security against undetectable online dictionary attacks.

## 1. Introduction

Authenticated key exchange is one of the most fundamental problems in cryptography and network security. In 1992, Bellovin and Merritt [1] introduced encrypted key exchange (or EKE) protocols, which allow

*arbitrary two parties, who share only a low-entropy password, to establish a common high-entropy secret key (called a session key) over an insecure public network. *

Since the work of Bellovin and Merritt [1], password-only authenticated key exchange (PAKE) protocols have attracted much greater attention mainly due to the persistent popularity of passwords as a practical (and cheap) authentication method [2]. Since the publication of the first EKE protocol (with only heuristic security arguments), many provably secure PAKE protocols have been published. Recent examples include the protocol of Katz and Vaikuntanathan [3], which enjoys both round optimality and provable security in the standard model (i.e., without random oracles and ideal ciphers).

A major challenge in designing PAKE protocols is to protect passwords from a* dictionary attack*, in which an adversary enumerates all possible passwords while testing each one against known password verifiers in order to determine the correct one. The design of two-party PAKE protocols secure against dictionary attacks has been extensively studied over the past two decades and is now fairly well understood. However, three-party PAKE protocols have received far less attention and preventing dictionary attacks is more challenging in the three-party setting. Unlike the two-party setting that assumes the same password is shared between the two parties, the three-party setting assumes that the two parties (commonly known as* clients*) wishing to establish a session key do not share the same password but hold their individual password shared only with a trusted server. This implies that in the three-party setting, a malicious client can attempt to mount an insider dictionary attack against its partner client. Indeed, many published three-party PAKE protocols were subsequently found to be vulnerable to an insider online/offline dictionary attack (e.g., [4–10]).

It is widely regarded that the design of key exchange protocols (including PAKE protocols) is notoriously hard, and conducting security analysis for such protocols is time-consuming and error-prone [11–13]. The many flaws discovered in published protocols have promoted the use of formal models and rigorous security proofs [14–16]. In the provable security paradigm for protocol analysis, a deductive reasoning process is adopted whereby emphasis is placed on a proven reduction from the problem of breaking the protocol to another problem believed to be computationally hard. A complete mathematical proof with respect to cryptographic definitions provides a strong assurance that a protocol is behaving as desired. It is now standard practice for protocol designers to provide security proofs in a well-defined formal model in order to assure protocol implementers about the security properties of protocols.

Over the past decade, we have seen a number of PAKE protocols proposed in the three-party setting [4–8, 17–29]. Many of these published protocols either did not have a proof of security [5, 6, 17, 22–25] or were subsequently found to be flawed [4–10, 12, 23, 24, 27, 30–36]. There are only a handful of provably secure three-party PAKE protocols [4, 7, 8, 21] whose claimed security properties have not been invalidated. However, there are limitations in the security proof of these protocols. For example, the protocols of [7, 8] are proven secure in the random oracle model. Although a proof of security in the random oracle model is definitely better than having no proof, it may not guarantee security in the real world (currently an open question). The protocols of [4, 21] are proven secure in a restricted model where the adversary is not allowed to corrupt protocol participants. Note that a protocol proven secure in such a restricted model cannot guarantee its security against attacks by malicious clients including insider online/offline dictionary attacks. (Readers who are unfamiliar with formal security models are referred to Section 2.1.) Although Yang and Cao [37] proposed a new three-party key exchange protocol that was proven secure in the standard model, the protocol is based on the ElGamal encryption scheme and thus requires a server's public key as well as clients' passwords to be preestablished before the protocol is ever executed. We refer the readers to [33, 38–44] for other recently published protocols designed to work in a “hybrid” setting where a cryptographic key is required in addition to passwords.

To the best of our knowledge, there is no published three-party PAKE protocol whose security is proven secure in the standard model that allows an adversary to corrupt protocol participants. In this work, we present the first three-party PAKE protocol that achieves provable security in the standard model against an active adversary with the corruption capability. We prove the security of session keys for our protocol in the widely accepted indistinguishability-based model of Bellare, Pointcheval, and Rogaway [14]—this model is, probably, one of the most popular proof models in the provable security paradigm for key exchange protocols. However, the indistinguishability-based security of session keys proven in the Bellare-Pointcheval-Rogaway model (and several other standard models) does not imply the security of passwords against undetectable online dictionary attacks, in which each guess on the password is checked undetectably via an online transaction with the server (see Section 2.3 for more details). We address this problem by providing a separate proof of security for the protocol against undetectable online dictionary (UDOD) attacks. This second proof is compact and elegant and does not rely upon idealized assumptions about the cryptographic primitives.  Table 1 compares our protocol against other provably secure three-party PAKE protocols in terms of security proofs.

The remainder of this paper is structured as follows.  Section 2 describes a formal proof model along with the associated definitions of security.  Section 3 presents our proposed three-party PAKE protocol. In Section 4, we prove that the proposed protocol achieves not only the typical indistinguishability-based security of session keys but also the password security against undetectable online dictionary attacks. We conclude the paper in Section 5.

## 2. Formal Setting

In this section, we

1. first describe a security model adapted from the Bellare-Pointcheval-Rogaway 2000 model [14],
2. define a typical indistinguishability-based security of session keys, which we call the* SK security*,
3. provide a simple and intuitive definition of security against undetectable online dictionary attacks.

### 2.1. The Security Model

*Protocol Participants*. Let *𝒞* be the set of all clients registered with the trusted authentication server *S*. Clients *C*, *C*′ ∈ *𝒞* who are both registered with *S* may run a three-party PAKE protocol *P* at any point in time to establish a session key. Let *𝒰* = *𝒞* ∪ {*S*}. A party *U* ∈ *𝒰* may have several instances involved in distinct, possibly concurrent, executions of protocol *P*. We use Π_*U*_^*i*^ to denote the *i*th instance of party *U*. A client instance Π_*C*_^*i*^ is said to* accept* when it successfully computes its session key sk_*C*_^*i*^ in a protocol execution.

*Long-Term Keys*. Each client *C* ∈ *𝒞* chooses a password pw_*C*_ from a fixed dictionary   PW and shares it with the server *S* via a secure channel. Accordingly, *S* holds all the passwords {pw_*C*_ | *C* ∈ *𝒞*}. Each password pw_*C*_ is used as the long-term secret key of *C* and *S*.

*Partnership*. The notion of partnership is a key element in defining the security of the protocol. Two instances are* partners* if both participate in a protocol execution and establish a (shared) session key. We define the partnership relations between instances using the notions of session identifiers and partner identifiers (see [45] on the role and the possible construct of session and partner identifiers as a form of partnering mechanism that enables the right session key to be identified in concurrent protocol executions.). A session identifier (sid) is a unique identifier of a protocol session and is defined as a function of the messages transmitted in the protocol session. We use sid_*U*_^*i*^ to denote the  sid of instance Π_*U*_^*i*^. A partner identifier (pid) is the set of participants of a specific protocol session. Instances should receive as input a  pid before they can run the protocol. By pid_*U*_^*i*^, we denote the  pid given to instance Π_*U*_^*i*^. Notice that pid_*C*_^*i*^ consists of three participants: server *S*, client *C*, and another client *C*′ with whom Π_*C*_^*i*^ believes it runs the protocol. We say that any two instances Π_*C*_^*i*^ and Π_*C*′_^*j*^ are* partners* if (1) both Π_*C*_^*i*^ and Π_*C*′_^*j*^ have accepted, (2)  *sid*_*C*_^*i*^ = *sid*_*C*′_^*j*^, and (3)  *pid*_*C*_^*i*^ = *pid*_*C*′_^*j*^.

*Adversary*. In the model, the probabilistic polynomial-time (ppt) adversary, *𝒜*, controls all the communications that take place between parties via a predefined set of oracle queries. For example, the adversary can ask participants to reveal session keys and passwords using  Reveal and  Corrupt queries as described below.

- Execute(Π_*C*_^*i*^, Π_*C*′_^*j*^, Π_*S*_^*k*^). This query models passive eavesdropping of a protocol execution. It prompts an honest execution of the protocol between the instances Π_*C*_^*i*^, Π_*C*′_^*j*^ and Π_*S*_^*k*^. The transcript of the protocol execution is returned as the output of the query.
- Send(Π_*U*_^*i*^, *m*). This query models active attacks against the protocol. It sends message *m* to instance Π_*U*_^*i*^ and returns the message that Π_*U*_^*i*^ sends out in response to *m*. A query of the form  Send(Π_*C*_^*i*^,  start : (*C*, *C*′, *S*)) prompts Π_*C*_^*i*^ to initiate the protocol with *pid*_*C*_^*i*^ = (*C*, *C*′, *S*).
- Reveal(Π_*C*_^*i*^). This query returns the session key sk_*C*_^*i*^. This query captures the notion of known key security (and it is often reasonable to assume that the adversary will be able to obtain session keys from any session different from the one under attack). Any client, Π_*C*_^*i*^, upon receiving such a query and if it has accepted and holds some session key, will send this session key back to *𝒜*. However, the adversary is not allowed to ask this query if it has already made a  Test query to the instance Π_*C*_^*i*^ or its partner instance (see below for explanation of the  Test oracle).
- Corrupt(*U*). This query captures not only the notion of forward secrecy but also unknown key share attacks and insider attacks. The query provides the adversary with *U*'s password pw_*U*_. Notice that a  Corrupt query does not result in the release of the session keys since the adversary already has the ability to obtain session keys through  Reveal queries. If *U* = *S* (i.e., the server is corrupted), all clients' passwords stored by the server will be returned.
- Test(Π_*C*_^*i*^). This query is the only oracle query that does not correspond to any of the adversary's abilities. If  Π_*C*_^*i*^ has accepted with some session key and is being asked a  Test(Π_*C*_^*i*^) query, then depending on a randomly chosen bit *b*, the adversary is given either the actual session key (when *b* = 1) or a session key drawn randomly from the session key distribution (when *b* = 0). The adversary can access the  Test oracle as many times as necessary. All the queries to the oracle are answered using the same value of the hidden bit *b*. Namely, the keys returned by the  Test oracle are either all real or all random. But, we require that for each different set of partners, the adversary should access the  Test oracle only once.

We represent the number of queries used by an adversary as an ordered sequence of five nonnegative integers, *Q* = (*q*_ex_, *q*_se_, *q*_re_, *q*_co⁡_, *q*_te_), where the five elements refer to the numbers of queries that the adversary made, respectively, to its  Execute,   Send,   Reveal,   Corrupt, and   Test oracles. We call this usage of queries by an adversary the* query complexity* of the adversary.

### 2.2. Session Key (SK) Security

We now define the basic security, called the SK security, of a 3-party PAKE protocol. As usual, we define the SK security via the notion of* freshness*. Intuitively, a fresh instance is one that holds a session key which should not be known to the adversary *𝒜*, and an unfresh instance is one whose session key (or some information about the key) can be known by trivial means. The formal definition of freshness is explained in Definition 1.

**Definition 1**

An instance Π_*C*_^*i*^ is fresh if none of the following occurs: (1) *𝒜* queries   Reveal(Π_*C*_^*i*^) or   Reveal(Π_*C*′_^*j*^), where Π_*C*′_^*j*^ is the partner of Π_*C*_^*i*^ and (2) *𝒜* queries *Corrupt*(*U*), for some *U* ∈ *pid*_*C*_^*i*^, before Π_*C*_^*i*^ or its partner Π_*C*′_^*j*^ accepts.

The SK security of a 3-party PAKE protocol *P* is defined in the context of the following two-stage experiment.

**Stage 1**

*𝒜* makes any oracle queries at will except that:

- *𝒜* is not allowed to ask the  Test(Π_*C*_^*i*^) query if the instance Π_*C*_^*i*^ is unfresh.
- *𝒜* is not allowed to ask the  Reveal(Π_*C*_^*i*^) query if it has already made a  Test query to Π_*C*_^*i*^ or Π_*C*′_^*j*^, where Π_*C*′_^*j*^ is the partner of Π_*C*_^*i*^.

**Stage 2**

Once *𝒜* decides that Phase 1 is over, it outputs a bit *b*′ as a guess on the hidden bit *b* chosen by the   Test oracle. *𝒜* is said to succeed if *b* = *b*′.

Let   Succ be the event that *𝒜* succeeds in this experiment. Then we define the advantage of *𝒜* in breaking the SK security of protocol *P* as

(1) $$\begin{array}{c} {Adv}_{P}^{\text{sk}}\left(𝒜\right)=2\cdot \text{Pr}\left[Succ\right]-1,\\ {Adv}_{P}^{\text{sk}}\left(t,Q\right)=\underset{𝒜}{\max}\left\{{Adv}_{P}^{\text{sk}}\left(𝒜\right)\right\}, \end{array}$$

where the maximum is over all   ppt adversaries *𝒜* with time complexity at most *t* and query complexity at most *Q*.

**Definition 2**

A 3-party PAKE protocol *P* is* SK-secure* if, for any ppt adversary *𝒜* asking at most *q*_se_ Send queries, *Adv*_*P*_^sk^(*𝒜*) is only negligibly larger than *c* · *q*_se_/|*PW*|, where *c* is a very small constant (usually around 2 or 4) when compared with |*PW*|.

### 2.3. Modelling Undetectable Online Dictionary Attacks

The SK security does not imply security against undetectable online dictionary attacks. In other words, a 3-party PAKE protocol that is not secure against an undetectable online dictionary attack may be rendered SK-secure. To see this, suppose that a 3-party PAKE protocol *P* is susceptible to undetectable online dictionary attacks whereby an attacker *A* can find out the password of any registered client *B*. Then, we can construct an adversary *𝒜* who attacks protocol *P* with advantage 1 as follows.

*Corruption.* If *A* is a registered client, *𝒜* queries   Corrupt(*A*) to obtain the password pw_*A*_. Otherwise, *𝒜* skips this step.

*Undetectable Online Dictionary Attacks.* Next, *𝒜* runs the protocol *P* in the same way as *A* conducts its undetectable online dictionary attacks against client *B*. Note that *𝒜* can perfectly simulate *A*'s attack by using the disclosed password pw_*A*_ and by asking oracle queries appropriately. At the end of this step, *𝒜* will obtain the password pw_*B*_ as a result of the attacks.

*Impersonation. 𝒜* then initiates a new protocol session by querying   Send(Π_*C*_^*i*^,   start:(*B*, *C*, *S*)), where Π_*C*_^*i*^ is an unused instance of an uncorrupted client *C*. *𝒜* runs this session as per the protocol specification, but simulating by itself all the actions of *B* (by using pw_*B*_). At the end of the session, the instance Π_*C*_^*i*^ will accept with its session key sk_*C*_^*i*^.

*Test.* The instance Π_*C*_^*i*^ is fresh as (1) no   Reveal query has been made on Π_*C*_^*i*^ or its partner (which does not exist) and (2) no   Corrupt query has been made against any of *B*, *C*, and *S*. Thus, *𝒜* may ask the   Test(Π_*C*_^*i*^) query. Since *𝒜* can compute the session key sk_*C*_^*i*^ by itself, it follows that Pr_*P*,*𝒜*_[*Succ*] = 1 and thus *Adv*_*P*_^sk^(*𝒜*) = 1.

Since verifying the correctness of a password guess may require more than one   Send queries to be asked, *𝒜* may have to ask   Send queries as many times as *d* · |*PW*|, for some integer *d* ≥ 1, to correctly determine the password pw_*A*_. Then, even if *Adv*_*P*_^sk^(*𝒜*) = 1, the following holds for some *c* ≥ 1:

(2) $$\begin{array}{c} {Adv}_{P}^{\text{sk}}\left(𝒜\right)\leq \frac{cd\left|PW\right|}{\left|PW\right|}, \end{array}$$

and the protocol *P* is rendered SK-secure by Definition 2.

This result is not surprising since we call a protocol SK-secure if mounting an online dictionary attack by asking   Send queries is the best an adversary can do. However, we want to be able to distinguish undetectable online dictionary attacks from detectable online dictionary attacks, and ensure that the best an adversary can do is to mount a detectable online dictionary attack. The following new definitions together provide a simple and intuitive way of capturing security against undetectable online dictionary attacks.

**Definition 3**

The   Send(Π_*S*_^*k*^, *m*) query models an* online dictionary attack* if both the following are true at the time of the termination of instance Π_*S*_^*k*^: (1) *m* was not output by a previous   Send query asked to an instance of *C* by which, Π_*S*_^*k*^ believes, *m* was sent and (2) the adversary *𝒜* queried neither   Corrupt(*S*) nor   Corrupt(*C*).

In Definition 3, the first condition implies that a straightforward delivery of a message between instances is not considered as an online dictionary attack while the second condition implies that, when *C*′ is the (assumed) peer of client *C*, the adversary *𝒜* can corrupt the peer client *C*′ to mount an (insider) online dictionary attack. Note that our definition of an online dictionary attack does not impose any restriction on asking   Reveal queries.

Consider the two-stage experiment described in the previous section. Let   Undet be the event that in the experiment, a server instance terminates normally when an online dictionary attack was mounted against the instance. We say that the adversary *𝒜* succeeds in mounting an undetectable online dictionary attack if the event   Undet occurs.

**Definition 4**

A 3-party PAKE protocol *P* is secure against an undetectable online dictionary attack if, for any ppt adversary *𝒜* asking at most *q*_se_ Send queries, Pr_*P*,*𝒜*_[Undet] is only negligibly larger than *c* · *q*_se_/|*PW*|, where *c* is as in Definition 2.

## 3. Our Proposed Protocol

As we have earlier claimed, our proposed protocol presented in this section is the first three-party PAKE protocol proven secure in the standard model against an active adversary who has the corruption ability.

### 3.1. Preliminaries

We begin by reviewing some cryptographic primitives which underlie the security of our protocol.

*Decisional Diffie-Hellman (DDH) Assumption.* Let *𝔾* be a cyclic (multiplicative) group of prime order *q*. Since the order of *𝔾* is prime, all the elements of *𝔾*, except 1, are generators of *𝔾*. Let *g* be a random fixed generator of *𝔾* and let *x*, *y*, *z* be randomly chosen elements in *ℤ*_*q*_ where *z* ≠ *xy*. Informally stated, the DDH problem for *𝔾* is to distinguish between the distributions of (*g* ^*x*^, *g* ^*y*^, *g* ^*xy*^) and (*g* ^*x*^, *g* ^*y*^, *g* ^*z*^), and the DDH assumption is said to hold in *𝔾* if it is computationally infeasible to solve the DDH problem for *𝔾*. More formally, we define the advantage of *𝒟* in solving the DDH problem for *𝔾* as *Adv*_*𝔾*_^ddh^(*𝒟*) = |Pr[*𝒟*(*𝔾*, *g*, *g* ^*x*^, *g* ^*y*^, *g* ^*xy*^) = 1] − Pr[*𝒟*(*𝔾*, *g*, *g* ^*x*^, *g* ^*y*^, *g* ^*z*^) = 1]|. We say that the DDH assumption holds in *𝔾* if *Adv*_*𝔾*_^ddh^(*𝒟*) is negligible for all ppt algorithms *𝒟*. We denote by *Adv*_*𝔾*_^ddh^(*t*) the maximum value of *Adv*_*𝔾*_^ddh^(*𝒟*) over all algorithms *𝒟* running in time at most *t*.

*Message Authentication Codes.* Let Σ = (*Gen*, *Mac*, *Ver*) be a message authentication code (MAC) scheme. The key generation algorithm   Gen takes as input a security parameter 1^*ℓ*^ and outputs a key *k* chosen uniformly at random from {0,1}^*ℓ*^. The MAC generation algorithm   Mac takes as input a key *k* and a message *m* and outputs a MAC (also known as a tag) *σ*. The MAC verification algorithm   Ver takes as input a key *k*, a message *m*, and a MAC *σ* and outputs 1 if *σ* is valid for *m* under *k* or outputs 0 if *σ* is invalid. Let *Adv*_Σ_^euf-cma^(*𝒜*) be the probability that an adversary *𝒜* succeeds in breaking the existential unforgeability of Σ under adaptive chosen message attacks. We say that the MAC scheme Σ is secure if *Adv*_Σ_^euf-cma^(*𝒜*) is negligible for every  ppt adversary *𝒜*. We use *Adv*_Σ_^euf-cma^(*t*, *q*_mac_, *q*_ver_) to denote the maximum value of *Adv*_Σ_^euf-cma^(*𝒜*) over all  ppt adversaries *𝒜* running in time at most *t* and asking at most *q*_mac_ and *q*_ver_ queries to its MAC generation and verification oracles, respectively.

*Two-Party PAKE Protocols.* Let  2PAKE be a two-party PAKE protocol that outputs session keys distributed in {0,1}^*ℓ*^. We assume that  2PAKE is SK-secure against an adversary who is given access to all the oracles:   Send,   Execute,  Reveal,   Corrupt, and   Test. Let  *Adv*_2PAKE_^sk^(*𝒜*) be the advantage of an adversary *𝒜* in breaking the SK security of  2PAKE. We require that, for all  ppt adversaries *𝒜* making at most *q*_se_ Send queries,  *Adv*_2PAKE_^sk^(*𝒜*) is only negligibly larger than *q*_se_/|PW|. We denote by ADV_2PAKE_^sk^(*t*, *Q*) the maximum value of Adv_2PAKE_^sk^(*𝒜*) over all  ppt adversaries *𝒜* with time complexity at most *t* and query complexity at most *Q*.

### 3.2. Protocol Description

Let *A* and *B* be two clients who wish to establish a session key, and let *S* be a trusted server with which *A* and *B* have secretly shared their respective passwords pw_*A*_ and pw_*B*_. Our protocol proceeds as follows.

**Step 1**

*A* and *S* establish a shared secret key *k*_*AS*_ by running the two-party protocol  2PAKE. Likewise, *B* and *S* establish a shared secret key *k*_*BS*_.

**Step 2**

*A* (resp., *B* and *S*) selects a nonce *n*_*A*_ (resp., *n*_*B*_ and *n*_*S*_) at random from *ℤ*_*q*_ and sends *A*||*n*_*A*_ (resp., *B*||*n*_*B*_ and *S*||*n*_*S*_) to the other two parties. All the parties (*A*, *B*, and *S*) define their session identifiers as  sid_*A*_ = sid_*B*_ = sid_*S*_ = *A*||*n*_*A*_||*B*||*n*_*B*_||*S*||*n*_*S*_.

**Step 3**

*A* chooses a random *x* ∈ *ℤ*_*q*_, computes *X* = *g* ^*x*^ and  *σ*_*AS*_ = Mac_*k*_*AS*__(*A*||*X*||sid_*A*_), and sends *A*||*X*||*σ*_*AS*_ to *S*. Meanwhile, *B* chooses a random *y* ∈ *ℤ*_*q*_, computes *Y* = *g* ^*y*^ and  *σ*_*BS*_ = Mac_*k*_*BS*__(*B*||*Y*||sid_*B*_), and sends *B*||*Y*||*σ*_*BS*_ to *S*.

**Step 4**

*S* checks that  Ver_*k*_*AS*__(*A*||*X*||sid_*S*_, *σ*_*AS*_) = 1 and  Ver_*k*_*BS*__(*B*||*Y*||sid_*S*_, *σ*_*BS*_) = 1. If either of these is untrue, *S* aborts the protocol. Otherwise, *S* computes  *σ*_*SA*_ = Mac_*k*_*AS*__(*S*||*Y*||sid_*S*_) and  *σ*_*SB*_ = Mac_*k*_*BS*__(*S*||*X*||sid_*S*_) and sends *S*||*Y*||*σ*_*SA*_ and *S*||*X*||*σ*_*SB*_ to *A* and *B*, respectively.

**Step 5**

*A* computes the session key sk = *Y* ^*x*^ if  Ver_*k*_*AS*__(*S*||*Y*||sid_*A*_, *σ*_*SA*_) = 1, while *B* computes the session key sk = *X* ^*y*^ if  Ver_*k*_*BS*__(*S*||*X*||sid_*B*_, *σ*_*SB*_) = 1. *A* and *B* abort the protocol if their verification fails.

**Figure 1:**
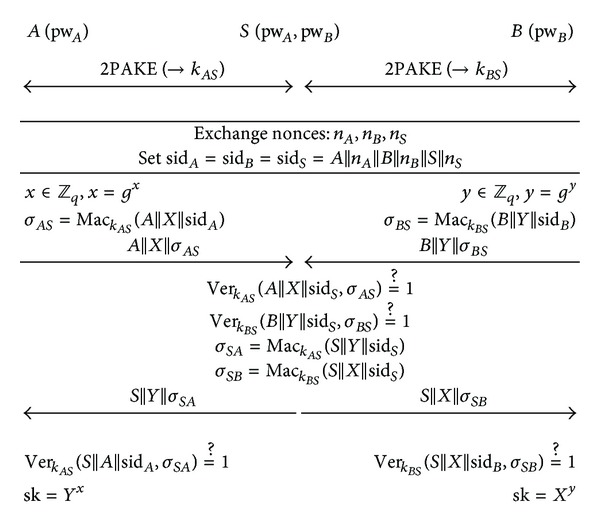
The proposed three-party PAKE protocol.

The operation of this protocol is illustrated in Figure 1. Steps 1 and 2 of the protocol are independent and can be performed in parallel. The session-key computation in the protocol is the same as in the Diffie-Hellman key exchange protocol (i.e., sk = *g* ^*xy*^). Hence, it is straightforward to verify the correctness of the protocol.

## 4. Security Proofs

In this section we prove that our three-party PAKE protocol is SK-secure and is resistant to undetectable online dictionary attacks. The proofs of both properties rely on neither random oracles nor ideal ciphers. Therefore, if   2PAKE is instantiated with a protocol proven secure in the standard model (e.g., [3, 49]), our three-party PAKE protocol would also be provably secure in the standard model. Hereafter, we denote our protocol by   3PAKEsm (“sm” for “standard model”).

### 4.1. Proof of SK Security

We first claim that, if the underlying two-party protocol   2PAKE is SK-secure, then the   3PAKEsm protocol is SK-secure as well under the DDH assumption in *𝔾* and the security of the MAC scheme Σ.

**Theorem 5**

Let *Q* = (*q*_ex_, *q*_se_, *q*_re_, *q*_co⁡_, *q*_te_). For any adversary with query complexity at most *Q* and time complexity at most *t*, its advantage in attacking protocol   3PAKEsm is bounded by

(3) Adv3PAKEsmsk(t,Q)≤2·Adv2PAKEsk(t′,Q′)+(qse+qex)2|𝔾| +2·qse·AdvΣeuf-cma(t′,4,4) +4·Adv𝔾ddh(t′),

where *Q*′ = (2*q*_ex_, *q*_se_, 0, *q*_co⁡_, 2*q*_ex_ + *q*_se_) and *t*′ is the maximum time required to perform an entire experiment involving an adversary who attacks protocol  3PAKEsm with time complexity *t*.

**Proof**

Assume an adversary *𝒜* attacking protocol   3PAKEsm with time complexity *t* and query complexity *Q* = (*q*_ex_, *q*_se_, *q*_re_, *q*_co⁡_, *q*_te_). We prove Theorem 5 by introducing a sequence of experiments **E** **x** **p** **r**_0_,…, **E** **x** **p** **r**_5_ and bounding the difference in *𝒜*'s advantage between two consecutive experiments. **E**x**p** **r**_0_ is the original experiment (described in Section 2.2) in which *𝒜* attacks the actual protocol, and **E** **x** **p** **r**_5_ is the experiment in which the advantage of *𝒜* is 0. Let  Succ_*i*_ be the event that *𝒜* correctly guesses the hidden bit *b* (chosen by the  Test oracle) in experiment **E** **x** **p** **r**_*i*_. By definition, we get  Adv_3PAKEsm_^sk^(*𝒜*) = 2 · Pr[Succ_0_] − 1.

Before providing details of the proof, we first define the notion of an* uncorrupted* instance.

**Definition 6**

We say an instance Π_*U*_^*i*^ is clean if no one in  pid_*U*_^*i*^ has been asked a   Corrupt query. Otherwise, we say it is unclean.

*Experiment* **E** **x** **p** **r**_1_. We modify the experiment so that each different MAC key is chosen uniformly at random from {0,1}^*ℓ*^ for all clean instances. The difference in *𝒜*'s success probability between **E** **x** **p** **r**_0_ and **E** **x** **p** **r**_1_ is bounded by

**Claim 1**

(4) $$\begin{array}{c} \left|\text{Pr}\left[{Succ}_{1}\right]-\text{Pr}\left[{Succ}_{0}\right]\right|\leq {Adv}_{2PAKE}^{\text{sk}}\left(t^{\prime },Q^{\prime }\right). \end{array}$$

**Proof**

Assume that the advantage of *𝒜* in attacking protocol   3PAKEsm is different between two experiments **E** **x** **p** **r**_0_ and **E** **x** **p** **r**_1_. Then we prove the claim by constructing, from *𝒜*, an adversary  *𝒜*_2PAKE_ attacking protocol   2PAKE with time complexity *t*′ and query complexity *Q*′.

*𝒜*_2PAKE_ begins by choosing a bit *b* uniformly at random.  *𝒜*_2PAKE_ then invokes *𝒜* as a subroutine and answers the oracle queries of *𝒜* on its own as follows.

Execute *Queries*.  *𝒜*_2PAKE_ answers   Execute queries of *𝒜* by making   Execute and   Test queries to its own oracles. Specifically,  *𝒜*_2PAKE_ handles each   Execute(Π_*A*_^*i*^, Π_*B*_^*j*^, Π_*S*_^*k*^) query as follows.

- If anyone in {*A*, *B*, *S*} has been corrupted, then  *𝒜*_2PAKE_ answers the   Execute query as in experiment **E** **x** **p** **r**_0_.
- Otherwise,  *𝒜*_2PAKE_ first makes two queries   Execute(Π_*A*_^*i*^, Π_*S*_^*k*^) and   Execute(Π_*B*_^*j*^, Π_*S*_^*k*′^). Let T_2PAKE_ and  T_2PAKE_′ be two transcripts returned in response to the   Execute queries. Next,  *𝒜*_2PAKE_ makes the queries   Test(Π_*A*_^*i*^) and   Test(Π_*B*_^*j*^) and receives in return two keys ${\bar{k}}_{AS}$ and ${\bar{k}}_{BS}$ (either real or random).  *𝒜*_2PAKE_ then generates the messages of Steps 2–4 of protocol   3PAKEsm, using ${\bar{k}}_{AS}$ and ${\bar{k}}_{BS}$ as the MAC keys. Finally,  *𝒜*_2PAKE_ returns these messages prepended by T_2PAKE_ and  T_2PAKE_′.

Send *Queries*. At a high level, the simulation of the   Send oracle is similar to that of the   Execute oracle. Specifically,  *𝒜*_2PAKE_ handles each   Send(Π_*U*_^*i*^, *m*) query as follows.

- If the instance Π_*U*_^*i*^ is clean or the message *m* belongs to Step 2 or later steps, then  *𝒜*_2PAKE_ answers the query as in experiment **E** **x** **p** **r**_0_.
- Otherwise,  *𝒜*_2PAKE_ answers it by making the same query to its own   Send oracle. If the query causes Π_*U*_^*i*^ to accept, then  *𝒜*_2PAKE_ also makes a   Test(Π_*U*_^*i*^) query (if it had not previously asked a   Test query to the partner of Π_*U*_^*i*^). As in the simulation of the   Execute oracle,  *𝒜*_2PAKE_ uses the output of this   Test query as the MAC key in generating the messages of Steps 2–4 of protocol   3PAKEsm.

Reveal *Queries*. These queries are answered in the obvious way. Namely,  *𝒜*_2PAKE_ responds to the query   Reveal(Π_*C*_^*i*^) by returning the session key sk_*C*_^*i*^.

Corrupt *Queries*. When *𝒜* queries   Corrupt(*U*),  *𝒜*_2PAKE_ makes the same query to its own   Corrupt oracle and simply forwards the output to *𝒜*.

Test *Queries*.  *𝒜*_2PAKE_ answers these queries according to the bit *b* chosen at the beginning of the simulation. That is,  *𝒜*_2PAKE_ returns real session keys, which it has computed on its own, if *b* = 1, and otherwise returns random keys chosen uniformly at random from *𝔾*.

Now at some point in time, when *𝒜* terminates and outputs its guess *b*′,  *𝒜*_2PAKE_ outputs 1 if *b* = *b*′ and outputs 0 otherwise.

From the simulation above, it is easy to see that  *𝒜*_2PAKE_ has at most time complexity *t*′ and query complexity *Q*′. The advantage of  *𝒜*_2PAKE_ in attacking protocol   2PAKE is immediate if we notice the following.

- The probability that  *𝒜*_2PAKE_ outputs 1 when its   Test oracle returns real session keys is equal to Pr[Succ_0_], the probability that *𝒜* correctly guesses the bit *b* in experiment **E** **x** **p** **r**_0_.
- The probability that  *𝒜*_2PAKE_ outputs 1 when its   Test oracle returns random keys is equal to Pr[Succ_1_], the probability that *𝒜* correctly guesses the bit *b* in experiment **E** **x** **p** **r**_1_.

This means that  Adv_2PAKE_^sk^(*𝒜*_2PAKE_) =|Pr[Succ_1_] − Pr[Succ_0_]|.  Claim 1 then follows.

*Experiment* **E** **x** **p** **r**_2_. Let   Repeat be the event that a nonce selected by an instance of a party is selected again by another instance of the same party. The experiment **E** **x** **p** **r**_2_ is aborted, and the adversary does not succeed, if the event   Repeat occurs. This is the only difference between **E** **x** **p** **r**_1_ and **E** **x** **p** **r**_2_. By a straightforward calculation, we get the following.

**Claim 2**

(5) $$\begin{array}{c} \left|\text{Pr}\left[{Succ}_{2}\right]-\text{Pr}\left[{Succ}_{1}\right]\right|\leq \frac{{\left(q_{\text{se}}+q_{\text{ex}}\right)}^{2}}{2\left|𝔾\right|}. \end{array}$$

*Experiment* **E** **x** **p** **r**_3_. Let   Forge be the event that the adversary *𝒜* makes a   Send query of the form   Send(Π_*U*_^*i*^, *V*||∗||*σ*) before querying   Corrupt(*W*), for some  *W* ∈ pid_*U*_^*i*^, such that (1) *σ* is a valid tag on *V*||∗||sid_*U*_^*i*^ and (2) no oracle had not previously generated a tag on *V*||∗||sid_*U*_^*i*^. If  Forge occurs, this experiment is aborted and the adversary does not succeed. Then we have the following.

**Claim 3**

(6) $$\begin{array}{c} \left|\text{Pr}\left[{Succ}_{3}\right]-\text{Pr}\left[{Succ}_{2}\right]\right|\leq q_{\text{se}}\cdot {Adv}_{\Sigma }^{\text{euf-cma}}\left(t^{\prime },4,4\right). \end{array}$$

**Proof**

Assuming that the event   Forge occurs, we construct, from *𝒜*, an algorithm *ℱ* who outputs, with a nonnegligible probability, a forgery against the MAC scheme Σ. The algorithm *ℱ* is given oracle access to  Mac_*k*_(·) and  Ver_*k*_(·). The goal of *ℱ* is to produce a message/tag pair (*m*, *σ*) such that (1) *σ* is a valid tag on the message *m* (i.e.,  Ver_*k*_(*m*, *σ*) = 1) and (2) *ℱ* had not previously queried its oracle  Mac_*k*_(·) on the message *m*.

Let *n* be the number of all active sessions that *𝒜* initiates by asking a   Send query. First, *ℱ* chooses a random *α* ∈ {1,…, *n*}. *ℱ* then simulates the oracle calls of *𝒜* as in experiment **E** **x** **p** **r**_2_; except that in the *α*th session, it answers   Send queries by accessing its MAC generation and verification oracles. If   Forge occurs in the *α*th session, *ℱ* halts and outputs the message/tag pair generated by *𝒜* as its forgery. Otherwise, *ℱ* halts and outputs a failure indication. This simulation is perfect unless the adversary *𝒜* makes a   Corrupt query against a participant of the *α*th session. But note that the event of *𝒜* making such a   Corrupt query should not happen if   Forge occurs in the *α*th session.

From the simulation, it is immediate that  *Adv*_Σ_^euf-cma^(*ℱ*) = Pr⁡[*Forge*]/*n*. Since *n* ≤ *q*_se_, we get   Pr[*Forge*] ≤ *q*_se_ · *Adv*_Σ_^euf-cma^(*ℱ*). Then, Claim 3 follows by noticing that *ℱ* has at most time complexity *t*′ and makes at most 4 queries to   *Mac*_*k*_(·) and   *Ver*_*k*_(·).

*Experiment* **E** **x** **p** **r**_4_. This experiment is different from **E** **x** **p** **r**_3_ in that the session key sk of each pair of instances partnered via an   Execute query is chosen uniformly at random from *𝔾* instead of being computed as sk = *g* ^*xy*^ = *X* ^*y*^ = *Y* ^*x*^. As the following claim states, the difference in *𝒜*'s advantage between **E** **x** **p** **r**_3_ and **E** **x** **p** **r**_4_ is negligible if the DDH assumption holds in *𝔾*.

**Claim 4**

(7) $$\begin{array}{c} \left|\text{Pr}\left[{Succ}_{4}\right]-\text{Pr}\left[{Succ}_{3}\right]\right|\leq {Adv}_{𝔾}^{\text{ddh}}\left(t^{\prime }\right). \end{array}$$

**Proof**

Assume that the advantage of *𝒜* is nonnegligibly different between **E** **x** **p** **r**_3_ and **E** **x** **p** **r**_4_. We prove the claim by constructing, from *𝒜*, a distinguisher *𝒟* that solves the DDH problem in *𝔾*. Let (*g*_1_, *g*_2_, *g*_3_) ∈ *𝔾* ^3^ be an instance of the DDH problem given as input to *𝒟*. *𝒟* begins by choosing a bit *b* uniformly at random. *𝒟* then invokes *𝒜* as a subroutine and proceeds to simulate the oracles. *𝒟* answers all the oracle queries of *𝒜* as in experiment **E** **x** **p** **r**_3_, except that it handles each   Execute(Π_*A*_^*i*^, Π_*B*_^*j*^, Π_*S*_^*k*^) query by

1. selecting two random *a*_*i*_, *b*_*i*_ ∈ *ℤ*_*q*_,
2. computing *X*′ = *g*_1_^*a*_*i*_^ and *Y*′ = *g*_2_^*b*_*i*_^,
3. returning a transcript generated with *X*′ and *Y*′ in place of *X* and *Y*,
4. then setting sk_*A*_^*i*^ = sk_*B*_^*j*^ = *g*_3_^*a*_*i*_*b*_*i*_^.

Let *b*′ be the output of *𝒜*. *𝒟* outputs 1 if *b* = *b*′ and outputs 0, otherwise.

Then, the following is clear:

- The probability that *𝒟* outputs 1 on a true Diffie-Hellman triple is exactly the probability that *𝒜* correctly guesses the bit *b* in experiment **E** **x** **p** **r**_3_.
- The probability that *𝒟* outputs 1 on a random triple is exactly the probability that *𝒜* correctly guesses the bit *b* in experiment **E** **x** **p** **r**_4_.

This completes the proof of Claim 4.

*Experiment* **E** **x** **p** **r**_5_. In this experiment, the session key sk_*C*_^*i*^ of each instance Π_*C*_^*i*^ activated by a   Send query is chosen uniformly at random from *𝔾* if no one in  *pid*_*C*_^*i*^ has been corrupted before Π_*C*_^*i*^ determines its session identifier   *sid*_*C*_^*i*^. The difference in *𝒜*'s advantage between **E** **x** **p** **r**_4_ and **E** **x** **p** **r**_5_ is bounded by the following.

**Claim 5**

(8) $$\begin{array}{c} \left|\text{Pr}\left[{Succ}_{5}\right]-\text{Pr}\left[{Succ}_{4}\right]\right|\leq {Adv}_{𝔾}^{\text{ddh}}\left(t^{\prime }\right). \end{array}$$

**Proof**

The proof of this claim is essentially similar to that of Claim 4. From the adversary *𝒜* whose advantage is nonnegligibly different between **E** **x** **p** **r**_4_ and **E** **x** **p** **r**_5_, we construct a distinguisher *𝒟* that solves the DDH problem in *𝔾*. Let (*g*_1_, *g*_2_, *g*_3_) ∈ *𝔾* ^3^ be an instance of the DDH problem given as input to *𝒟*. *𝒟* begins by selecting a bit *b* uniformly at random and generating a list   DDHList which is used to link an instance of the DDH problem to a session identifier, *𝒟* then runs *𝒜* as a subroutine and simulates the oracles. It handles all the queries of *𝒜* as in experiment **E** **x** **p** **r**_4_ except for   Send queries.

Consider a query of the form   *Send*(Π_*C*_^*i*^, *U*||*n*_*U*_) which delivers a random nonce *n*_*U*_ to instance Π_*C*_^*i*^. Whenever such a query is made, *𝒟* answers it as follows.

- If *n*_*U*_ is not the last nonce that Π_*C*_^*i*^ is expected to receive, *𝒟* simply waits for the next nonce.
- Otherwise, *𝒟* defines   *sid*_*C*_^*i*^ and checks that anyone in   *pid*_*C*_^*i*^ was corrupted.
  - If so, *𝒟* responds to the query as in experiment **E** **x** **p** **r**_4_.
  - If not, *𝒟* checks if the list   DDHList contains an entry of the form   (*sid*_*C*_^*i*^, *X*′, *Y*′, *Z*′), where *X*′, *Y*′, *Z*′ ∈ *𝔾*.
    - If it does, *𝒟* computes   *σ*_CS_ = *Mac*_*k*_CS__(*C*||*Y*′||*sid*_*C*_^*i*^) and returns *C*||*Y*′||*σ*_CS_ in response to the query.
    - Otherwise, *𝒟* selects two random *a*_*i*_, *b*_*i*_ ∈ *ℤ*_*q*_, computes *X*′ = *g*_1_^*a*_*i*_^, *Y*′ = *g*_2_^*b*_*i*_^, *Z*′ = *g*_3_^*a*_*i*_*b*_*i*_^, and   *σ*_CS_ = *Mac*_*k*_CS__(*C*||*X*′||*sid*_*C*_^*i*^), returns *C*||*X*′||*σ*_CS_ to *𝒜*, and finally adds the tuple   (*sid*_*C*_^*i*^, *X*′, *Y*′, *Z*′) to   DDHList.

When *𝒜* makes a  Send query that causes an instance Π_*C*_^*i*^ to accept, *𝒟* checks if   DDHList contains an entry of the form  (sid_*C*_^*i*^, *X*′, *Y*′, *Z*′). If so, *𝒟* sets sk_*C*_^*i*^ = *Z*′. Otherwise, *𝒟* computes sk_*C*_^*i*^ as in experiment **E** **x** **p** **r**_4_. For all other  Send queries of *𝒜*, *𝒟* answers them as in experiment **E** **x** **p** **r**_4_. Now when *𝒜* terminates and outputs its guess *b*′, *𝒟* outputs 1 if *b* = *b*′ and outputs 0 otherwise.

One can easily see the following.

- The probability that *𝒟* outputs 1 on a true Diffie-Hellman triple is exactly the probability that *𝒜* correctly guesses the bit *b* in experiment **E** **x** **p** **r**_4_.
- The probability that *𝒟* outputs 1 on a random triple is exactly the probability that *𝒜* correctly guesses the bit *b* in experiment **E** **x** **p** **r**_5_.

This implies Claim 5.

In experiment **E** **x** **p** **r**_5_, the session keys of all fresh instances are chosen uniformly at random from *𝔾* and thus the adversary *𝒜* obtains no information on the bit *b* chosen by the   Test oracle. Therefore, it follows that   Pr[*Succ*_5_] = 1/2. This result combined with the previous claims yields the statement of Theorem 5.

### 4.2. Proof of Resistance to Undetectable Online Dictionary Attacks

We now claim that   3PAKEsm is secure against undetectable online dictionary attacks as long as the   2PAKE protocol is SK-secure.

**Theorem 7**

Let   Undet be as defined in Section 2.3 and assume that for any ppt adversary *𝒜*′ asking at most *q*_se_ Send queries,  Adv_2PAKE_^sk^(*𝒜*′) is only negligibly larger than  *q*_se_/|PW|. Then, for any   ppt adversary *𝒜* asking at most *q*_se_ Send queries,  Pr_3PAKEsm,*𝒜*_[Undet] is only negligibly larger than  2 · *q*_se_/|PW|.

**Proof**

Let *𝒜* be a  ppt adversary who asks *q*_se_ Send queries in mounting an undetectable online dictionary attack against   3PAKEsm. Consider the experiment **E** **x** **p** **r**_1_ described in the proof of Theorem 5 (see Section 4.1). By  Undet_1_ (resp.,  Undet_0_), we denote the event   Undet defined in experiment **E** **x** **p** **r**_1_ (resp., **E** **x** **p** **r**_0_). We prove Theorem 7 by first proving Claim 6 and then Claim 7.

**Claim 6**

|Pr_3PAKEsm,*𝒜*_[Undet_1_] − Pr_3PAKEsm,*𝒜*_[Undet_0_]| is only negligibly larger than  *q*_se_/|PW|.

**Claim 7**

Pr_3PAKEsm,*𝒜*_[Undet_1_] is only negligibly larger than  *q*_se_ | PW|.

**Proof**

We prove the claim by constructing an adversary *𝒜*′ who attacks the SK security of   2PAKE witfh advantage equal to  |Pr_3PAKEsm,*𝒜*_[Undet_1_] − Pr_3PAKEsm,*𝒜*_[Undet_0_]|.

*𝒜*′ chooses a random bit *b* ∈ {0,1} and invokes the adversary *𝒜* as a subroutine. *𝒜*′ then simulates the oracles for *𝒜* in the exactly same way as in the simulation for the proof of Claim 1. *𝒜*′ outputs 1 if  Undet occurs and 0 otherwise. From the way the oracles are simulated, it is easy to see the following.

- The probability that *𝒜*′  outputs 1 when its   Test oracle returns real session keys is equal to the probability that the event   Undet occurs in experiment **E** **x** **p** **r**_0_.
- The probability that *𝒜*′ outputs 1 when its   Test oracle returns random keys is equal to the probability that the event   Undet occurs in experiment **E** **x** **p** **r**_1_.

Since *𝒜*′ makes at most *q*_se_ Send queries, we obtain the statement of Claim 6.

**Proof**

Assume that  Pr_3PAKEsm,*𝒜*_[Undet_1_] is nonnegligibly larger than  *q*_se_/|PW|. Given the adversary *𝒜*, we construct an adversary *𝒜*′ against   2PAKE who asks at most *q*_se_ Send queries but has an advantage nonnegligibly larger than  *q*_se_/|PW|.

*𝒜*′ runs *𝒜* as a subroutine while simulating the oracles on its own. *𝒜*′ handles all the oracle queries of *𝒜* as in the experiment **E** **x** **p** **r**_1_ except for   Send queries. When *𝒜* makes a   Send(Π_*U*_^*i*^, *m*) query, *𝒜*′ checks if *m* is a message for initiating a new session (of   3PAKEsm) or the   Send query belongs to an execution of   2PAKE.

1. If both are untrue, *𝒜*′ responds to the query as in experiment **E** **x** **p** **r**_1_.
2. Otherwise, *𝒜*′ answers it by making the same query to its own   Send oracle. If the query prompts Π_*U*_^*i*^ to accept, then *𝒜*′ checks if Π_*U*_^*i*^ is clean.
  - If so, *𝒜*′ sets the MAC key of Π_*U*_^*i*^ to be a random key drawn uniformly from {0,1}^*ℓ*^.
  - Otherwise, *𝒜*′ makes a   Reveal(Π_*U*_^*i*^) query and sets the MAC key of Π_*U*_^*i*^ to be the output of this   Reveal query.

Let Π_*S*_^*t*^ be any server instance against which *𝒜* has mounted an online dictionary attack. Let *k*_*S*_^*t*^ be the session key (i.e., the MAC key) that the instance Π_*S*_^*t*^ has computed in its execution of   2PAKE. In order for the instance Π_*S*_^*t*^ to terminate normally, the adversary *𝒜* has to make a query of the form   Send(Π_*S*_^*t*^, *C*||∗||*σ*_CS_) such that   *Ver*_*k*_*S*_^*t*^_(*C*||∗||*sid*_*S*_^*t*^, *σ*_CS_) = 1. When *𝒜* makes such a    Send query (i.e., when the event Undet_1_ occurs), *𝒜*′ makes a   Test query against the instance Π_*S*_^*t*^. Note that the instance Π_*S*_^*t*^ is fresh as (1) it is partnered with no instance and (2)  *S* and *C* must have not been corrupted. Let ${\bar{k}}_{S}^{t}$ be the key returned in response to the   Test query. *𝒜*′ outputs 1 if   ${Ver}_{{\bar{k}}_{S}^{t}}(C||*||{sid}_{S}^{t},\sigma_{\text{CS}})=1$ and outputs 0, otherwise. If  Undet_1_ does not occur, *𝒜*′ outputs a random bit. Then, it is not hard to see that

(9) Adv2PAKEsk(𝒜′)=2·Pr2PAKE,𝒜′[Succ]−1=2·(Pr3PAKEsm,𝒜[Undet1]   +12(1−Pr3PAKEsm,𝒜[Undet1]))−1=Pr3PAKEsm,𝒜[Undet1].

This completes the proof of Claim 7.

Theorem 7 immediately follows from Claims 6 and 7.

## 5. Conclusion

In this work, we have presented a three-party PAKE protocol whose security does not rely on the existence of random oracles. The model that we used to prove the security of our protocol allows the adversary to ask    Corrupt queries and thus captures insider attacks as well as forward secrecy. It is a known fact that proving the security of protocols in such a model is of particular importance in the three-party setting as insider dictionary attacks are most serious threats to three-party PAKE protocols. To the best of our knowledge, our protocol is the first three-party PAKE protocol proven secure against insider, active adversaries in the standard model (i.e., without random oracles and ideal ciphers). Another advantage our protocol has over previously published protocols is that it also achieves provable security against undetectable online dictionary attacks. The latter property is also significant as designing three-party PAKE protocol secure against undetectable online dictionary attacks is an ongoing challenge (as evidenced by the number of three-party PAKE protocols found to be vulnerable to an undetectable online dictionary attack). We leave it as a future work to design a three-party PAKE protocol that achieves not only provable security in the standard model but is more efficient than our protocol.

## Figures and Tables

**Table 1:** Security proof comparison.

Protocol	Idealized assumption	Adversary capability	Resistance to UDOD attacks^†^
Our protocol	None	Not restricted	Proven
GPAKE [21]	None	Restricted from corrupting parties	No [4]
NGPAKE [4]	None		Not proven
Lin and Hwang [7]	Random oracles	Not restricted	Not proven
Wu et al. [8]	Random oracles	Not restricted	Not proven

^†^Resistance to undetectable online dictionary attacks.
